# Oncologists Knowledge and Attitudes Towards Providing Dietary Guidance to Patients With Cancer

**DOI:** 10.1177/15598276251414349

**Published:** 2026-01-12

**Authors:** Shireen Kassam, Zahra Kassam, David Nemirovsky, Andriy Derkach, Susan Chimonas, Cynthia Thomson, Urvi A. Shah

**Affiliations:** 1 4616 Department of Haematology, King’s College London, London, UK (SK); 2 8629 Faculty of Health and Wellbeing, University of Winchester, Winchester, UK (SK); 3 Southlake Health, Stronach Regional Cancer Centre, Toronto, ON, Canada (ZK); 4 Department of Radiation Oncology, 7938 University of Toronto, Toronto, ON, Canada (ZK); 5 Department of Epidemiology and Biostatistics, 5803 Memorial Sloan Kettering Cancer Center, New York, NY, USA (DN, AD); 6 Center for Health Policy and Outcomes, 5803 Memorial Sloan Kettering Cancer Center, New York, NY, USA (SC); 7 613590 Department of Health Promotion Sciences, University of Arizona Cancer Center (UACC), Tucson, AZ, USA (CT); 8 Department of Medicine, 5803 Memorial Sloan Kettering Cancer Center, New York, NY, USA (UAS)

**Keywords:** oncology, dietary counselling, nutrition education, cancer survivorship, plant-based diet, clinical practice patterns, physician attitudes

## Abstract

**Background:** Multiple studies link dietary patterns to cancer risk and survivorship outcomes with cancer-specific guidelines focusing on fiber and plant-rich, minimally processed diets. Additionally, patients frequently report unmet needs for dietary counselling. There is limited data on oncologists’ knowledge and attitudes towards this evidence and whether it influences their clinical practice. **Methods:** A 25-question survey was distributed to oncology professionals with 150 evaluable responses. The survey assessed respondents’ demographics, personal dietary choices, knowledge of dietary guidelines, and practice behaviors. Responses were analyzed using descriptive statistics and Fisher’s exact and Pearson’s Chi-squared tests. **Results:** Most respondents considered dietary choices important for cancer risk reduction (77.4%), during treatment (66.7%) and for survivorship (76.6%), with 23.3% referring all patients to a dietitian. Barriers to implementing dietary counselling included lack of time (66.7%) or knowledge (54%), or resources (54.7%) or lack of reimbursement (22%). Oncologists following plant-based dietary patterns were more likely to value dietary counselling, engage in self-directed learning, and perceive diet as relevant throughout the cancer care continuum. **Conclusions:** Despite established dietary guidance, significant gaps in training and practice persist. Enhancing nutrition education, increasing access to resources, consistent reimbursement of dietitian appointments, and generating robust clinical evidence are essential to support oncologists.

“Dietary habits are associated with cancer risk and survival, whilst also affecting the risk of other chronic diseases, including those associated with cancer risk.”

## Introduction

Dietary habits are associated with cancer risk and survival, whilst also affecting the risk of other chronic diseases, including those associated with cancer risk. Estimates vary, but the landmark report by Doll and Peto from 1981 and revisited in 2015 suggested that up to 35% of cancers could be attributable to diet although the risk varies based on cancer site.^1,2^ More conservative calculations from the United States suggest that 3.4% and 4.9% of cancer risk in women and men, respectively, are associated with dietary risk factors.^3^ When you include alcohol consumption as a risk factor, this increases to almost 10%. Unhealthy dietary patterns are now also the leading risk factor for chronic illness and premature death worldwide.^4^ Globally, diets have increasingly shifted towards being high in ultra-processed and animal-based foods while lacking in fiber-rich plant foods.^4^ This type of diet promotes inflammation, insulin resistance, and adversely affects the gut microbiome, which may promote carcinogenesis.^5^ Studies show that these unhealthy dietary patterns negatively impact rates of remission, survival, and quality of life after a cancer diagnosis whilst increasing the risk of cardiometabolic diseases and second cancers.^6,7^

This knowledge has been incorporated into national and international guidance from the World Cancer Research Fund (WCRF)/American Institute of Cancer Research (AICR) and American Cancer Society (ACS) on cancer prevention and survivorship with recommendations to increase intake of fruits, vegetables, whole grains, and beans, whilst avoiding processed meat and alcohol, and minimizing red meat and ultra-processed foods.^8-10^ Yet, awareness amongst people with cancer and their families remains low.^11^ This may be, in part, because the support from dietitians during cancer care is limited and mainly directed towards preventing weight loss.^12^ In addition, oncologists have little undergraduate or postgraduate nutrition training and are not equipped to address dietary questions.^13-15^ Patients with cancer are keen to receive dietary guidance from their oncologist, but rarely receive this, leading to patients obtaining their information from non-accredited sources.^11,12,16-19^

The aim of this study was to investigate: (1) Whether oncologists are aware of available dietary guidelines for cancer; (2) Whether oncologists routinely educate patients about dietary guidelines and support patients in making healthier dietary changes; (3) Oncologists’ attitudes and barriers toward the importance of dietary counselling for improving the health and well-being of their patients; and (4) Whether oncologists’ personal dietary choices influence their dietary recommendations for patients.

## Methods

### Survey and Study Design

A 25-question online survey was developed collaboratively between the research team, composed of a hematologist, a hematologist and medical oncologist, a radiation oncologist, a research dietitian, a sociologist, and a statistician, following a literature review to establish existing knowledge. The survey was divided into the following sections: General information about the respondent including demographics and clinical practice, respondents’ dietary behaviors, respondents’ current practice with regards to dietary counselling, attitudes and barriers towards dietary counselling, and knowledge of current dietary guidelines for cancer. The full survey can be found in the supplemental data file 1.

### Participant Inclusion and Recruitment

The survey was administered to oncology providers through the American Society of Clinical Oncology (ASCO) Research Survey Pool (RSP), a service available to members who engage in survey research. The RSP provides a mechanism for surveying oncology professionals made available through ASCO’s Center for Research and Analytics (CENTRA). The survey “pool” consists of ASCO members worldwide who have opted-in to participate in optional research survey projects conducted by other members. The survey was administered using an online survey tool from Qualtrics. The survey was distributed to 1157 current members between July 9th and August 20th, 2024, selected at random by CENTRA. The following selections were made from the ASCO database of members; fellows and currently practicing oncologists (exclude students); Stage of career: currently practicing (exclude retired); Degrees: advanced practice providers and doctoral level physicians (exclude nurses); Location: domestic and international; Professional role: gynecologic oncologist/hematologist/medical oncologist/pediatric oncologist/radiation oncologist/surgical oncologist; Cancer type: all cancer types. In addition, the survey was distributed to members of the British Society of Haematology (BSH). An initial question was included to exclude those not currently seeing patients with cancer for at least 4 h per week from completing the survey. No incentive was offered to respondents.

### Ethics and Data Protection

This study was reviewed by the Memorial Sloan Kettering Cancer Center Institutional Review Board and determined to be exempt from further review. The survey was fully anonymized.

### Statistics

Descriptive analyses are presented for responses to survey questions. Fisher’s exact and Pearson’s Chi-squared tests were used to analyze associations between participants’ personal dietary patterns and their practice behaviors, attitudes and beliefs. Participants were divided into 3 groups: omnivorous, plant-based dietary patterns (pescatarian/vegetarian/vegan/plant-based/Mediterranean), and Western diet based on their self-reported diet pattern.

## Results

A total of 155 individuals completed the survey. Five were excluded as they did not undertake a minimum of 4 h of clinical practice per week. In the final analysis, 150 (96.8%) respondents were included (Table 1). Respondents included 128 (85.3%) ASCO members and 22 (14.7%) BSH members. Most participants identified as White (38%) or Asian/Asian American (41%), were aged 30–45 years (66%), practiced in USA (32.7%), and were fully accredited physicians (73.3%). Primary specialties included medical oncology (58%), malignant hematology (15.3%), or both (14%). Most respondents were practicing primarily in a university/academic setting (56%) and practiced equally in inpatient and outpatient settings (50.7%). The most frequently treated cancer sites included breast (41.3%), general cancer (36.7%) and hematologic malignancies (21%).

**Table 1.** Respondents Characteristics.

Characteristic		N (%)
Member society	American Society of Clinical Oncology	128 (85.3)
	British Society of Haematology	22 (14.7)
Gender	Male	76 (50.6)
	Female	74 (49.3)
Racial/ethnic group	Asian or Asian American	62 (41.3)
	White	57 (38)
	Hispanic/Latino	13 (8.7)
	Black or African American	8 (5.3)
	Middle Eastern or North African	8 (5.3)
	Not answered	2 (1.3)
Age	<30 years	7 (4.7)
	30–45 years	99 (66)
	46–60 years	36 (24)
	>60 years	8 (5.3)
Country of practice	USA	49 (32.7)
	UK/Ireland	22 (14.7)
	India	19 (12.7)
	South America	9 (6)
	Africa	7 (4.7)
	Philippines	5 (3.3)
	South-East Asia	5 (3.3)
	Pakistan	4 (2.7)
	Canada	3 (2)
	Other	27 (18)
Current career stage	Fully accredited physician	110 (73.3)
	Resident/fellow/registrar	40 (26.7)
Specialty	Medical oncology	87 (58)
	Malignant hematology	23 (15.3)
	Hematology and medical oncology	21 (14)
	Radiation oncology	9 (6)
	Surgical oncology	9 (6)
	Palliative care	1 (0.7)
Practice type	Academic/university	84 (56)
	Community/private	46 (30.7)
	Equal time in academic and community	16 (10.7)
	Not applicable	4 (2.7)
Practice location	Equal time in inpatient and outpatient	76 (50.7)
	Outpatient	62 (41.3)
	Inpatient	8 (5.3)
	Not applicable	4 (2.7)
Primary cancer site (select all that apply)	Hematology	32 (21)
	Breast	62 (41.3)
	General cancer	55 (36.7)
	Gastrointestinal	36 (24)
	Genitourinary/gynecology	31 (20.7)
	Thoracic	17 (11.3)
	Neurologic, soft tissue, bone, or skin	15 (10)
	Head and neck	11 (7.3)
	Pediatric	6 (4)
Dietary patterns of respondents	Omnivore diet with minimal processed/junk foods	48 (32)
	Western diet (a diet typical in the UK, USA, and Canada, tends to be higher in processed foods and animal products and lower in plant-foods)	34 (22.7)
	Mediterranean (primarily plant-based, emphasizing whole grains, olive oil, fruits, vegetables, beans, legumes and nuts, with minimal animal proteins preferring fish and seafood).	31 (20.7)
	Lacto-ovo vegetarian (vegetarian diet with eggs and dairy)	12 (8)
	Plant-based (minimal or occasional consumption of animal foods)	4 (2.7)
	Pescatarian (vegetarian diet with seafood)	2 (1.3)
	Low carbohydrate/ketogenic (high fat diet with low carbohydrates (<25% calories or <100 g/day))	1 (0.7)
	Not answered	18 (12)
Education on nutrition and cancer	No, I have not received specific training. I taught myself through reading books or journal articles	70 (46.7)
	No, I have not received specific training, nor have I spent time reading about this topic	40 (26.7)
	Yes, I received formal training during medical school	8 (5.3)
	Yes, I received formal training during residency	7 (4.7)
	Yes, I received formal training during oncology training	8 (5.3)
	I have taken an accredited course or attended a conference on nutrition and health	17 (11.3)

N, number of respondents.

### Respondents’ Practice Patterns

Just under a quarter of respondents referred all their patients with cancer to a dietitian/nutritionist while 1.3% referred none of their patients. Respondents also mentioned referring patients for reasons such as patient request (46%), cancer symptoms or treatment side effects that affect nutritional status (58%), and chronic comorbidities such as obesity, diabetes mellitus, cardiovascular disease (20%). When asked about the frequency of discussing diet and nutrition with patients, responses included very often (24%), often (34.7%), sometimes (23.3%), rarely (6%), and not applicable (12%).

Respondents were asked if they provided available cancer-specific dietary guidelines to their patients. While 47.4% provided guidelines, 17.3% were not aware of any guidelines and 35.3% discussed nutrition but did not provide any guidelines. The guidelines most often used included National Comprehensive Cancer Network (NCCN), ASCO, WCRF/AICR, and ACS (see supplemental file 2, Table 1).

### Respondents’ Dietary Guidance

Most respondents considered dietary choices “very important” or “somewhat important” for reducing cancer risk (77.4%), during cancer treatment (66.7%), and for survivorship/relapse prevention (76.6%) but less so for palliative treatment (55.3%) (Table 2). Dietary patterns considered most suitable to recommend to patients at all stages by respondents included Mediterranean (48.7%) or plant-based dietary patterns (40%) while only 9.3% felt low-carbohydrate diets should be recommended. Importantly, 30% of respondents felt that there is not enough evidence to recommend any dietary pattern (Table 3).

**Table 2.** Respondents Views on Importance of Dietary Choices.

	Very important	Somewhat important	Not sure/neutral	Somewhat unimportant	Very unimportant	Not answered
Prediagnosis/risk reduction	82 (54.7)	34 (22.7)	7 (4.7)	5 (3.3)	2 (1.3)	20 (13.3)
During curative treatment	64 (42.7)	36 (24)	17 (11.3)	9 (6)	4 (2.7)	20 (13.3)
Post treatment survivorship/relapse prevention	74 (49.3)	41 (27.3)	7 (4.7)	5 (3.3)	3 (2)	20 (13.3)
During palliative treatment	53 (35.3)	30 (20)	17 (11.3)	18 (12)	12 (8)	20 (13.3)

**Table 3.** Respondents’ Dietary Recommendations for Patients With Cancer.^a^

Recommendation	N (%)
Mediterranean	73 (48.7)
Plant-based diets (predominantly unprocessed plant-based foods; may or may not include small amounts of animal products)	60 (40)
There is not enough evidence to recommend any dietary pattern	48 (32)
Low-carbohydrate diets, including ketogenic and Paleo	14 (9.3)
Pescatarian	12 (8)
Other dietary pattern	3 (2)
Not answered for dietary pattern	20 (13.3)
Minimize/avoid processed meat	68 (45.3)
Minimize/avoid red meat	51 (34)
Increase fruits and vegetables	99 (66)
Increase whole grains	60 (40)
Increase beans and other legumes	52 (34.6)
Minimize/avoid sugary beverages	69 (46)
Minimize/avoid processed food	73 (48.7)
Eat high calorie foods or whatever you like to avoid losing weight	41 (27.3)
Eat more animal protein	10 (6.7)
Eat more plant protein	25 (16.7)
Eat more protein of any type (animal or plant)	49 (32.7)
Eat low-carbohydrate foods	19 (12.7)
Minimize dairy intake	7 (4.7)
Eat more dairy	11 (7.3)
Consider intermittent fasting	7 (4.7)
Eat foods to reduce weight to an ideal body weight	12 (8)
Doesn’t matter, eat what you want	25 (16.7)

^a^Select all that apply.

Table 3 shows specific dietary recommendations provided to patients by respondents. The most common recommendations given were to increase consumption of fruits and vegetables (66%), whole grains (40%), beans and legumes (34.6%), and protein (32.7%) and to avoid/minimize processed food (48.7%), sugar-sweetened beverages (46%), and processed meat (45.3%).

Regarding alcohol consumption, 43% respondents advised avoiding alcohol consumption, whereas 27% advised drinking in moderation (≤1 drink per day for women or ≤2 drinks per day for men) as acceptable, 11% didn’t provide advice, 7% provided individualized advice, and 12% did not respond to this question.

### Respondents’ Education, Attitudes, Barriers, and Knowledge

Only 15.3% of respondents reported that they had received formal nutrition education during medical school, residency or through oncology training. The duration of training varied from 1 to >20 h. Of the 84.7% of respondents that had not received formal nutrition education during training, 55.1% reported self-directed learning through reading books and journal articles and 13.4% reported attending an accredited course or conference. Despite this, 68% participants “strongly agreed” or “agreed” that it is the role of an oncologist/hematologist to discuss nutrition although numerous barriers were identified. The most frequent barriers were limited time (66.7%), lack of resources (55.3%) and lack of knowledge (54%) (Table 4). In addition, lack of reimbursement for dietary counselling was cited by 22% of respondents.

**Table 4.** Respondents Perceived Barriers When Discussing Nutrition With Patients.^a^

Barrier	N (%)
Lack of time in the clinical visit	100 (66.7)
Lack of resources to share with patients	83 (55.3)
Lack of my own knowledge/formal education about this topic	81 (54)
Too few outpatient dietitians	50 (33.3)
No outpatient dietitians	36 (24)
Not reimbursed by insurance	33 (22)
Lack of support from leadership	19 (12.7)

^a^Select all that apply.

Table 5 shows whether respondents believe that currently available evidence is sufficient to provide specific nutrition counselling and guidance to their patients at various stages of their cancer journey. While most respondents believe that dietary patterns can reduce comorbidities (72.7%) or improve quality of life (61.3%), fewer respondents believe it can improve treatment response (29.3%) or cancer-specific outcomes (26%).

**Table 5.** Respondents Beliefs as to Whether There is Sufficient Evidence to Provide Nutrition Recommendations to Patients.

	Yes	No	Unsure	Not answered
Reduce comorbidities (such as obesity, diabetes, and cardiovascular disease) to improve survival.	109 (72.7)	10 (6.7)	11 (7.3)	20 (13.3)
Improve quality of life	92 (61.3)	16 (10.7)	22 (14.7)	20 (13.3)
Improve cancer treatment response	44 (29.3)	57 (38)	29 (19.3)	20 (13.3)
Improve cancer-specific survival endpoints such as progression-free survival and overall survival	39 (26)	57 (38)	34 (22.7)	20 (13.3)

Respondents were asked about the type of evidence they would require in order to recommend a specific dietary pattern to their patients. The majority would like to see data from interventional clinical trials in cancer populations while a smaller proportion felt that epidemiologic, retrospective, preclinical data as well as data in non-cancer comorbidities would be acceptable to share. For interventional clinical trials, 66.7% would like to have evidence from a large, randomized phase 3 interventional dietary trial with a clinical cancer outcome as a primary endpoint; 64.7% evidence from a large, randomized phase 3 interventional dietary trial with a non-cancer clinical outcome (such as quality of life) as a primary endpoint; 44% evidence from a randomized phase 1 or 2 dietary interventional trial showing potential benefit and 25.3% evidence from a non-randomized phase 1 or 2 dietary interventional trial showing potential benefit. For non-trial evidence, 50% of participants would be satisfied with evidence from large population-based epidemiologic studies and preclinical data, 31.3% with evidence from retrospective studies with correlative data, and 34.7% with evidence from research confirming benefits of dietary changes for comorbidities (diabetes, cardiovascular disease, obesity).

### Respondents’ Personal Self-Reported Dietary Behaviors

Survey respondents’ dietary patterns mainly included minimally processed omnivorous diet (32%), standard Western diet (22.7%), and predominantly plant-based patterns such as Mediterranean diet (20.7%), lacto-ovo-vegetarian (8%), plant-based (minimal or occasional consumption of animal foods 2.7%), and pescatarian (1.3%) (Table 1). Only a small number of participants consumed fruits (8.7%), vegetables (8.7%), and whole grains (7.3%) three or more times per day (Table 6). Respondents estimated that a median of 50% (range 5–99%) of their diet was composed of unprocessed plant foods with 47.3% of respondents stating that this was ≥50% of their total consumed diet. Respondents also estimated that a median of 30% (0–90%) of their diet came from unprocessed animal foods, 5% (0–40%) from processed meat, and 10% (0–50%) from ultra-processed foods and drinks.

**Table 6.** Respondents Frequency of Consumption of Food Groups N (%).

	Never	<1 times weekly	1-2 times weekly	3-6 times weekly	1-2 times daily	≥3 times daily	Not answered
Whole fruit	1 (0.7)	10 (6.7)	29 (19.3)	33 (22)	46 (30.7)	13 (8.7)	18 (12)
Vegetables/leafy greens	0	2 (1.3)	17 (11.3)	45 (30)	55 (36.7)	13 (8.7)	18 (12)
Whole grains	3 (2)	19 (12.7)	28 (18.7)	34 (22.7)	37 (24.7)	11 (7.3)	18 (12)
Eggs	8 (5.3)	23 (15.3)	56 (37.3)	33 (22)	11 (7.3)	1 (0.7)	18 (12)
Seafood	22 (14.7)	25 (16.7)	58 (38.7)	19 (12.7)	7 (4.7)	1 (0.7)	18 (12)
Poultry	19 (12.7)	17 (11.3)	46 (30.7)	42 (28)	6 (4)	2 (1.3)	18 (12)
Dairy	3 (2)	10 (6.7)	21 (14)	41 (27)	46 (31)	11 (7.3)	18 (12)
Plant-based dairy alternatives	16 (10.7)	35 (23.3)	23 (15.3)	33 (22)	19 (12.7)	6 (4)	18 (12)
Plant protein	1 (0.7)	13 (8.7)	48 (32)	44 (29.3)	20 (13.3)	6 (4)	18 (12)
Red or processed meat	32 (21.3)	47 (31.3)	35 (23.3)	13 (8.7)	5 (3.3)	0	18 (12)
Ultra-processed foods and refined grains	7 (4.7)	40 (26.7)	45 (30)	28 (18.7)	9 (6)	3 (2)	18 (12)
Sugar-sweetened beverages	35 (23.3)	39 (26)	25 (16.7)	17 (11.3)	15 (10)	1 (0.7)	18 (12)

### Respondents’ Personal Choices May Affect Dietary Guidance Provided to Patients

In a subgroup analysis, responses were compared based on respondents’ own dietary choices between omnivorous (n = 42), Western (n = 32), and predominantly plant-based dietary patterns (n = 49) (Supplemental file 2, Table 2). While the frequency of discussing nutrition with their patients was not different, those following a Western diet were significantly more likely to tell patients to “eat what they want” (*P* = .005) and less likely to recommend low-carbohydrate diets (*P* = .049) when compared to those following an omnivorous or plant-based dietary pattern. Participants following a plant-based dietary pattern (36.7%) were more likely to consider their role to discuss nutrition with patients compared to participants following an omnivorous (23.9%) or Western diet pattern (15.6%) (*P* = .024). While the number of respondents receiving training was low overall, those following an omnivorous (14.2%) or plant-based (4.1%) diet were more likely to select that they received formal lectures during oncology training compared to Western diet (0%) (*P* = .036). Consistently, those following a plant-based diet (12.2%) were more likely to have taken an accredited course or attended a conference on nutrition compared to western/omnivorous diet (0%) (*P* = .01). Participants following a plant-based dietary pattern were more likely to consider diet to be “very important” for reducing the risk of cancer (*P* = .002), during curative treatment (*P* = .002), for survivorship/relapse prevention (*P* = .024), and during palliative treatment (*P* = .008) compared to those following an omnivorous or Western diet.

## Discussion

This survey highlights the current awareness amongst oncologists of the role of dietary patterns in cancer care as well as the persistent barriers that limit the integration of nutrition counselling into routine oncology practice. While the majority of respondents acknowledged the importance of dietary choices across the cancer continuum, from prevention to survivorship, there remains a significant gap between awareness and implementation. Only a subset of oncologists routinely provided cancer-specific dietary guidance, and many report inadequate training and resource constraints. This is at odds with patient expectations and their clinical needs given that prior studies have documented the importance placed on nutrition counselling by patients and the high prevalence of diet-related problems.^11,12^ Surprisingly, the percentage of respondents referring all their patients to a dietitian was higher than expected (23.3%), given that prior survey data have shown that staffing of nutrition and dietetic services in cancer centers is inadequate.^20^

Consistent with existing literature, the majority of respondents had received little or no formal nutrition education during their medical training, despite evidence that patients increasingly seek nutritional advice from their oncology care teams.^13,14,21^ This lack of training, coupled with structural barriers such as time limitations and insufficient access to dietetic support, was reported as a major impediment to offering comprehensive nutrition counselling. These findings underscore the need for enhanced nutrition education in medical curricula and greater institutional support for continuing medical education support in nutritional oncology as well as system-level changes to allow integration of dietary guidance into oncology services. This is being addressed to some degree with countries such as the USA and UK having issued guidance on nutrition training and competencies for medical students.^22,23^ Medical schools and residency programs are beginning to incorporate various types of nutrition programs including “food as medicine” and “culinary medicine” and postgraduate fellowships in nutrition are emerging.^24^ In addition, the American Board of Lifestyle Medicine offers a global certification program for physicians that includes nutrition education.^25^ Importantly, these training programs should integrate physician dietary counselling and skills development to build self-efficacy for adherence to plant-based eating patterns.

In fact, this study identified a relationship between oncologists’ self-reported personal dietary behaviors and their clinical practice. Respondents adhering to a plant-based dietary pattern (pescatarian/vegetarian/vegan/Mediterranean) were more likely to counsel patients on diet and engage in self-directed learning on nutrition. Conversely, respondents following a Western dietary pattern were less likely to consider dietary choices important at the various stages of a patient’s cancer journey. Respondents following plant-based dietary patterns were more likely to consider dietary choices important for patients receiving curative treatment. This correlation suggests that personal health behaviors may influence clinicians’ perceptions of the relevance of dietary advice in oncology, aligning with previously observed trends in Lifestyle Medicine.^26^ Given that fiber-rich dietary patterns are recommended in cancer-specific dietary guidelines, this may be a reflection of respondents’ own knowledge.^8-10^

While the majority of respondents expressed belief in the value of dietary interventions for improving quality of life and reducing comorbidities, fewer felt confident in the evidence linking diet to treatment response or cancer-specific outcomes. This is despite the presence of numerous observational studies and some recent clinical trials that suggest potential benefits of dietary interventions for cancer-specific outcomes.^27-31^

A significant proportion of respondents indicated that large randomized controlled trials with clinical endpoints would increase their confidence in recommending specific dietary patterns. These views are in keeping with the ASCO guideline on diet during cancer treatment, which states that the evidence supporting dietary interventions is currently limited.^32^ This underscores the need for further research to build a robust evidence-base and translate findings into clinical practice guidelines. Randomized studies are ongoing and being reported with encouraging results^33^. The LEANer trial demonstrated higher rates of pathological complete remission in patients with breast cancer on chemotherapy who received a nutrition and exercise intervention.^30^ The currently funded ENICTO U01 will address diet and relative dose intensity in 2 patient groups—those with breast cancer (≥65 years) and ovarian cancer.^34^

Strengths of this study include that it addresses an important yet under-explored area in oncology practice, especially given the degree of patient interest. It uniquely correlates oncologists’ personal dietary patterns with their clinical behaviors and attitudes, offering novel insight into how lifestyle factors may influence professional practice. The international sample offers insights across various oncology subspecialties and practice settings and provides actions that can be taken within the profession to address gaps in knowledge and practice. However, the study has a number of limitations. The small sample size limits the generalizability of the results although the 13% response rate is in keeping, if not better than other survey’s administered through ASCO.^15^ In addition, all of the participants were physicians, whereas nurse specialists, physician associates, and other allied health professionals play an important role in supporting patients through their cancer journey and are potentially more likely to be involved in assessing non-medical needs such as nutrition.^35^ There is also the potential that self-selection bias may have led to an overrepresentation of individuals with a greater interest in diet and nutrition. For example, 32.7% reported consuming a plant-based dietary pattern, a proportion that is higher than expected from a physician community.^36^ Additionally, responses were self-reported without the use of a validated dietary questionnaire and hence subject to recall and social desirability bias. Self-reported dietary patterns did not necessarily align with reported consumption of food group. For example, less than 10% of respondents consumed 3 or more portions of fruit, vegetables, and whole grains a day, yet 20% reported consuming a Mediterranean-style dietary pattern. Finally, while the study included participants from multiple countries, the majority were practicing in high-income settings, which may limit generalizability to low-resource contexts.

Lastly, barriers to dietary guidance include lack of resources. In order to implement system-wide changes, institutions will need to acquire more staffing in dietetics and include registered dietitians with oncology practice certification. Additionally, institutions will need to address patient barriers to adoption of healthy diet patterns such as food insecurity, knowledge deficits, and self-efficacy to achieve dietary goals.

## Conclusions

The results of this study emphasize the current state of awareness among oncologists of the importance of diet in cancer care, while also revealing critical gaps in training, confidence, and practice. There is a clear need for improved nutrition education throughout medical training, including support for enhancement of physician diet quality, as well as better integration of dietary guidance into routine oncology practice. Efforts to strengthen the evidence base through high-quality clinical trials, combined with system-level support and resource development, are essential to meet patient needs and realize the potential of dietary interventions in improving cancer outcomes.

## Supplemental Material


Supplemental Material - Oncologists Knowledge and Attitudes Towards Providing Dietary Guidance to Patients With Cancer
Supplemental Material for Oncologists Knowledge and Attitudes Towards Providing Dietary Guidance to Patients With Cancer by Shireen Kassam, Zahra Kassam, David Nemirovsky, Andriy Derkach, Susan Chimonas, Cynthia Thomson, and Urvi A. Shah in American Journal of Lifestyle Medicine.

Supplemental Material - Oncologists Knowledge and Attitudes Towards Providing Dietary Guidance to Patients With Cancer
Supplemental Material for Oncologists Knowledge and Attitudes Towards Providing Dietary Guidance to Patients With Cancer by Shireen Kassam, Zahra Kassam, David Nemirovsky, Andriy Derkach, Susan Chimonas, Cynthia Thomson, and Urvi A. Shah in American Journal of Lifestyle Medicine.
