# Supplementary material for: Oncologists Knowledge and Attitudes Towards Providing Dietary Guidance to Patients With Cancer
Source: Am J Lifestyle Med. 2026 Jan 12:15598276251414349. Online ahead of print. doi: 10.1177/15598276251414349 (PMC12799481; doi:10.1177/15598276251414349)
Supplement: Supplemental Material - Oncologists Knowledge and Attitudes Towards Providing Dietary Guidance to Patients With Cancer [file sj-pdf-1-ajl-10.1177_15598276251414349.pdf]

Knowledge and Attitudes of Practicing Oncologists Survey

Start of Block: Default Question Block

QID1 **Knowledge and Attitudes of Practicing Oncologists Survey**
A research team at Memorial Sloan Kettering Cancer Center in collaboration with University of Winchester and King's College Hospital, UK, and University of Toronto and Stronach Regional Cancer Centre, Canada is conducting a study about cancer and dietary practices. The goal is to establish the knowledge and attitudes of practicing oncologists on the availability of dietary evidence for cancer prevention and survival. In order to participate, you should have an active clinical practice with a minimum of 4 clinical hours per week. If you decide to participate, you will be asked to complete a 15-minute survey. All information that you provide will be strictly confidential, de-identified, and stored on a secure computer network. You can withdraw from the study at any time by closing the browser window. If you have any questions about the study, you may contact the principal investigator, **Urvi Shah, MD [**shahu@mskcc.org**] or the Center for Research and Analytics [**centra@asco.org**].** Thank you for your willingness to participate.

End of Block: Default Question Block

Start of Block: General information

QID48 **General Information**

QID2 Which of the following best describes you?

- ○ a. Male (1)
- ○ b. Female (2)
- ○ c. Non-binary (3)
- ○ d. Self-identify as: (4) ____
- ○ e. Prefer not to answer (5)

QID3 Which Racial/Ethnic Category best describes you? (select all that apply)

- a. American Indian or Alaska Native (1)
- b. Asian or Asian American (2)
- c. Black of African American (3)
- d. Hispanic or Latino (4)
- e. Native Hawaiian or Other Pacific Islander (5)
- f. Middle Eastern or North African (6)
- g. White (7)
- h. Self-identify as: (8) ____
- i. Prefer not to answer (9)

QID4 What is your current age?

- a. (1)
- b. 30-45 years (2)
- c. 46-60 years (3)
- d. >60 years (4)

QID5 In which country do you practice?

○ a. USA (1)

○ b. Canada (2)

○ c. UK/Ireland (3)

○ d. Other (please specify): (4)
____

QID6 What is your current career stage?

○ a. Medical Student (1)

○ b. Resident/Fellow/Registrar (2)

○ c. Advanced Practice Providers (Nurse Practitioners, Physician Assistants and Clinical Nurse Specialists) (3)

○ d. Fully accredited Physician (4)

○ e. Other (please specify): (5)
____

QID7 What is your specialty?

- ◯ a. Medical Oncology (1)
- ◯ b. Radiation Oncology (2)
- ◯ c. Surgical Oncology (3)
- ◯ d. Clinical Oncology (UK) (4)
- ◯ e. Benign Hematology (5)
- ◯ f. Malignant Hematology (6)
- ◯ g. Hematology and Medical Oncology (7)
- ◯ h. Internal Medicine/Primary Care (8)
- ◯ i. Palliative Care (9)
- ◯ j. Other (please specify): (10) ____

End of Block: General information

Start of Block: Block 7

QID46 In which setting do you see the majority of your patients?

QID39

	Academic/University (1)	Community/Private (2)	Equal time in academic and community (3)
Practice Type (1)	◯	◯	◯

QID47

	Inpatient (1)	Outpatient (2)	Equal time in inpatient and outpatient (3)
Patient Location (1)	○	○	○

QID9 What is the cancer disease site you primarily treat? (Select all that apply (up to 3 choices))

▢ a. General cancer (1)

▢ b. General hematology (2)

▢ c. Breast cancer (3)

▢ d. Gastrointestinal (4)

▢ e. Genitourinary/Gynecologic (5)

▢ f. Thoracic cancer (6)

▢ g. Head and neck cancer (7)

▢ h. Neurologic cancers (8)

▢ i. Soft tissue and bone cancers (9)

▢ j. Pediatric (10)

▢ k. Hematologic Malignancies (11)

▢ l. Other(s) (please specify): (12) ____

End of Block: Block 7

Start of Block: Block 2

QID11 In order to participate, please confirm that you undertake a minimum of 4 hours of clinical practice per week.

- a. Yes (1)
- b. No (2)

End of Block: Block 2

Start of Block: Provider Dietary Behaviors

QID31 **Provider Dietary Behaviors**

QID10 What is your predominant dietary pattern for most meals?

- a. Western diet (a diet typical in the UK, USA and Canada, tends to be higher in processed foods and animal products and lower in plant-foods) (1)
- b. Omnivore diet with minimal processed/junk foods (2)
- c. Pescatarian (vegetarian diet with seafood) (3)
- d. Lacto-ovo vegetarian (vegetarian diet with eggs and dairy) (4)
- e. Vegan (no animal products) (5)
- f. Plant-based (minimal or occasional consumption of animal foods) (6)
- g. Low carbohydrate/ketogenic (high fat diet with low carbohydrates ((7)
- h. Mediterranean (primarily plant-based, emphasizing whole grains, olive oil, fruits, vegetables, beans, legumes and nuts, with minimal animal proteins preferring fish and seafood). (8)
- i. Other (please specify): (9) ____

QID12 Please select your average frequency of consumption of the following food groups over the past month.

	Never (1)	<1 time a week (2)	1-2 times a week (3)	3-6 times a week (4)	1-2 times daily (5)	≥3 times daily (6)
Whole fruits (not juices) (1)						
Vegetables and leafy greens (2)						
Whole grains (brown rice, oatmeal, quinoa, barley, whole grain bread, whole grain pasta, whole grain muffin). Do not count white rice, white bread or refined muffins/cookies/pancakes. (3)						
Eggs (4)						
Seafood (fish, shellfish) (5)						
Poultry (6)						
Dairy products (milk, cheese, yogurt) (7)						
Plant-based dairy products (soy, nut, seed, grain or coconut-based milk, cheeses and yogurt) (8)						
Plant proteins (beans, legumes, seeds, nuts, tofu, meat alternatives) (9)						
Red/processed meats (bacon, hot dogs, sausage, cured meats) (10)						
Junk/processed/refined/fried foods (fast foods, candies, cakes, muffins, white rice, white bread) (11)						

Sugary drinks/sweetened beverages (soda or carbonated soft drinks, sports drinks, energy drinks, fruit-flavored or powdered drinks, vitamin-enhanced drinks, sweetened tea or coffee drinks, chocolate milk, milk or milk alternatives with added sugar) (12)	○	○	○	○	○	○

---

*

QID17 Please estimate the average percentage of your diet for these 4 food groups (must total to 100%).
Unprocessed plant foods (fruits, vegetables, seeds, nuts grains, tofu, legumes, beans, plant milks) : _______ (1)
Unprocessed animal foods (fish, poultry, eggs, dairy and red meat) : _______ (2)
Processed meats (hot dogs, deli meats, ham, pepperoni, bacon, sausage) : _______ (3)
Ultra-processed foods/drinks (Sugary drinks, fried snacks, desserts, and cakes) : _______ (4)
Total : ________

End of Block: Provider Dietary Behaviors

Start of Block: Provider Practice Behaviors

QID32 **Provider Practice Behaviors**

QID19 Which patients with cancer do you refer to a dietitian/nutritionist in your current practice? (Select all that apply)

- ☐ a. All patients with cancer (1)
- ☐ b. Patients that ask about diet or to see a nutritionist (2)
- ☐ c. Patients with cancer symptoms or treatment side-effects that affect nutritional status (3)
- ☐ d. Patients with chronic conditions such as obesity, type 2 diabetes, cardiovascular disease (4)
- ☐ e. None of my patients (5)

QID15 How often do you discuss diet and nutrition with your patients?

- ○ a. Very often (1)
- ○ b. Often (2)
- ○ c. Sometimes (3)
- ○ d. Rarely (4)
- ○ e. Never (5)

QID20 Do you base your nutrition recommendations for patients on any of the cancer-related dietary guidelines below? (Select all that apply)

- ☐ a. World Cancer Research Fund/American Institute for Cancer Research (1)
- ☐ b. NCCN guidelines (2)
- ☐ c. American Cancer Society (3)
- ☐ d. ASCO guidelines (4)
- ☐ e. Other (please specify): (5) ____
- ☐ f. I am not aware of any cancer-related dietary guidelines (6)
- ☐ g. I discuss nutrition but don’t give them any cancer-specific guidance or recommendations to follow (7)
- ☐ h. Not applicable - I don’t discuss nutrition with my patients (8)

QID21 What are your general dietary recommendations for the majority of your patients? (Select all that apply)

- ☐ a. Minimize/avoid processed meat (1)
- ☐ b. Minimize/avoid red meat (2)
- ☐ c. Increase fruits and vegetables (3)
- ☐ d. Increase whole grains (4)
- ☐ e. Increase beans and other legumes (5)
- ☐ f. Minimize/avoid sugary beverages (6)
- ☐ g. Minimize/avoid processed food (7)
- ☐ h. Eat high calorie foods or whatever you like to avoid losing weight (8)
- ☐ i. Eat more animal protein (9)
- ☐ j. Eat more plant protein (10)
- ☐ k. Eat more protein of any type (animal or plant) (11)
- ☐ l. Eat low carbohydrate foods (12)
- ☐ m. Minimize dairy intake (13)
- ☐ n. Eat more dairy (14)
- ☐ o. Consider intermittent fasting (15)
- ☐ p. Eat foods to reduce weight to an ideal body weight (16)
- ☐ q. Doesn't matter, eat what you want (17)
- ☐ r. Other (please specify): (18) ____

QID22 In general, what is your advice on alcohol use for your patients?

○ a. Drink in moderation – 1 drink or less per day for women or 2 drinks or less per day for men (1)

○ b. You advise patients to avoid alcohol as there is no safe limit for risk of cancer (2)

○ c. You do not provide specific advice (3)

○ d. Other (please specify): (4)

____

End of Block: Provider Practice Behaviors

Start of Block: Attitudes and Barriers

QID33 **Attitudes and Barriers**

QID23 It is the role of an oncologist/hematologist to discuss nutrition -

○ a. Strongly agree (1)

○ b. Agree (2)

○ c. Neither agree nor disagree (3)

○ d. Disagree (4)

○ e. Strongly disagree (5)

Skip To: QID25 If QID23 = a. Strongly agree

Skip To: QID25 If QID23 = b. Agree

Skip To: QID25 If QID23 = c. Neither agree nor disagree

QID24 If you answered disagree or strongly disagree to the prior question, what are the reasons? (Select all that apply)

- ▢ a. This role belongs to a dietitian or nurse (1)
- ▢ b. Discussing nutrition creates guilt in patients where they feel they caused their cancer (2)
- ▢ c. The data on nutrition and cancer is not robust enough (3)
- ▢ d. Discussing nutrition may cause worry or put extra pressure on patients (4)
- ▢ e. I don't feel prepared to sufficiently counsel patients on dietary modification (5)
- ▢ f. Other (please specify): (6) ____

QID25 What are the barriers you face when discussing nutrition with patients? (Select all that apply)

- ▢ a. Lack of my own knowledge/formal education about this topic (1)
- ▢ b. Lack of resources to share with patients (2)
- ▢ c. Lack of time in the clinical visit (3)
- ▢ d. No outpatient dietitians (4)
- ▢ e. Too few outpatient dietitians (5)
- ▢ f. Lack of support from leadership (6)
- ▢ g. Not reimbursed by insurance (7)
- ▢ h. Other (please specify): (8) ____

End of Block: Attitudes and Barriers

Start of Block: Knowledge

QID34 **Knowledge**

QID26 Which dietary patterns for cancer prevention/treatment/survivorship are based on current evidence and can be recommended to patients? (Select all that apply)

- ☐ a. Mediterranean (1)
- ☐ b. Pescatarian (2)
- ☐ c. Plant-based diets (predominantly unprocessed plant-based foods; may or may not include small amounts of animal products) (3)
- ☐ d. Low carbohydrate diets, including ketogenic and Paleo (4)
- ☐ e. There is not enough evidence to recommend any dietary pattern (5)
- ☐ f. Other (please specify): (6) ____

QID27 Have you received training on nutrition and cancer? (Select all that apply)

- ☐ a. Yes, I received formal training during medical school (1)
- ☐ b. Yes, I received formal training during residency (2)
- ☐ c. Yes, I received formal training during oncology training (3)
- ☐ d. Yes, I have taken an accredited course or attended a conference on nutrition and health (If yes, please specify course/conference) (4) ____
- ☐ e. No, I taught myself through reading books or journal articles (5)
- ☐ f. No, I have not received specific training, nor have I spent time reading about this topic (6)

Skip To: QID29 If QID27 = e. No, I taught myself through reading books or journal articles

Skip To: QID29 If QID27 = f. No, I have not received specific training, nor have I spent time reading about this topic

QID28 If you received formal training on nutrition and cancer, approximately how many total hours of training did you receive?

- a. 1-4 hours (1)
- b. 5-9 hours (2)
- c. 10-20 hours (3)
- d. >20 hours (4)

QID29 How important or unimportant are dietary choices for patients with cancer in the following situations?

	Very important (1)	Somewhat important (2)	Not sure/Neutral (3)	Somewhat unimportant (4)	Very unimportant (5)
Risk Reduction/Pre-diagnosis (1)	○	○	○	○	○
During curative treatment (2)	○	○	○	○	○
Post treatment survivorship/relapse prevention (3)	○	○	○	○	○
During palliative treatment (4)	○	○	○	○	○

QID37 If you believe there is currently insufficient evidence to recommend a specific dietary pattern to patients with cancer based on available evidence, what **minimum evidence** do you need before you will recommend this to patients?

	Yes (1)	No (2)	Unsure (3)
A large, randomized phase 3 interventional dietary trial with a clinical cancer outcome as a primary endpoint. (1)	○	○	○
A large, randomized phase 3 interventional dietary trial with a non-cancer clinical outcome (such as quality of life) as a primary endpoint. (2)	○	○	○
A randomized phase 1 or 2 dietary interventional trial showing potential benefit. (3)	○	○	○
A non-randomized phase 1 or 2 dietary interventional trial showing potential benefit. (4)	○	○	○
A negative randomized control trial but subgroup analysis shows survival benefit in the subgroup with high adherence. (5)	○	○	○
Large population based epidemiologic studies and preclinical data. (6)	○	○	○
Retrospective studies with correlative data. (7)	○	○	○
Research confirming benefits of dietary changes for comorbidities	○	○	○

(diabetes, cardiovascular disease, obesity). Therefore, you would extrapolate and recommend to patients with cancer with these comorbidities. (8)			
Other (please specify): (9)	○	○	○

QID38 Based on available evidence at this time do you believe there is enough information to provide nutrition recommendations to your patients for the following:

	Yes (1)	No (2)	Unsure (3)
Reduce comorbidities (such as obesity, diabetes, cardiovascular disease) to improve survival. (1)	○	○	○
Improve quality of life (2)	○	○	○
Improve cancer treatment response (3)	○	○	○
Improve cancer-specific survival endpoints such as progression-free survival and overall survival (4)	○	○	○

End of Block: Knowledge
