# Supplementary material for: Oncologists Knowledge and Attitudes Towards Providing Dietary Guidance to Patients With Cancer
Source: Am J Lifestyle Med. 2026 Jan 12:15598276251414349. Online ahead of print. doi: 10.1177/15598276251414349 (PMC12799481; doi:10.1177/15598276251414349)
Supplement: Supplemental Material - Oncologists Knowledge and Attitudes Towards Providing Dietary Guidance to Patients With Cancer [file sj-pdf-2-ajl-10.1177_15598276251414349.pdf]

Table 1. Do you base your nutrition recommendations for patients on any of the cancer-related dietary guidelines below? (Select all that apply)

	n (%)
I discuss nutrition but don't give them any cancer-specific guidance or recommendations to follow	53 (35.3)
I am not aware of any cancer-related dietary guidelines	26 (17.3)
World Cancer Research Fund/American Institute for Cancer Research	13 (8.7)
NCCN guidelines	42 (28)
American Cancer Society	13 (8.7)
ASCO guidelines	30 (20)
American Society for Parenteral and Enteral Nutrition (ASPEN) guidelines	1 (0.7)
Based on local departmental guidelines	3 (2)
Emphasize avoiding meat and processed foods	1 (0.7)
European Society for Medical Oncology (ESMO) guidelines	1 (0.7)
The European Society for Clinical Nutrition and Metabolism (ESPEN) guidelines	1 (0.7)
Based on local dietitian/nutritionist recommendations	3 (2)
Institut National du Cancer (INCA)	1 (0.7)
Personalized/based on my own recommendations	3 (2)

Table 2. Subgroup analysis comparing responses based on respondents own dietary choices

Characteristic	Omnivore<br>N = 42[1]	Pescatarian / Vegetarian / Vegan / Mediterranean<br>N = 49[1]	Western<br>N = 32[1]	p-value[2]
	Predominant Dietary Pattern			
**How often do you discuss diet and nutrition with your patients?**				0.2
a. Very often	12 (29%)	17 (35%)	3 (9.4%)	
b. Often	18 (43%)	18 (37%)	15 (47%)	
c. Sometimes	9 (21%)	12 (24%)	10 (31%)	
d. Rarely	3 (7.1%)	2 (4.1%)	4 (13%)	
**Selected Nutrition Recommendation Guidelines: I discuss nutrition but don't give them any cancer-specific guidance or recommendations to follow**	21 (50%)	15 (31%)	12 (38%)	0.2
**Selected Nutrition Recommendation Guidelines: I am not aware of any cancer-related dietary guidelines**	5 (12%)	11 (22%)	8 (25%)	0.3
**Selected Nutrition Recommendation Guidelines: World Cancer Research Fund/American Institute for Cancer Research**	4 (9.5%)	7 (14%)	0 (0%)	0.067
**Selected Nutrition Recommendation Guidelines: NCCN guidelines**	17 (40%)	15 (31%)	8 (25%)	0.3
**Selected Nutrition Recommendation Guidelines: American Cancer Society**	6 (14%)	5 (10%)	1 (3.1%)	0.3
**Selected Nutrition Recommendation**	14	9 (18%)	6	0.2

Characteristic	Predominant Dietary Pattern<br>Omnivore<br>N = 42[1]	 Pescatarian / Vegetarian / Vegan / Mediterranean<br>N = 49[1]	 Western<br>N = 32[1]	p-value[2]
**Guidelines: ASCO guidelines**	(33%)		(19%)	
**Selected Nutrition Recommendation Guidelines: Other (please specify):**	4 (9.5%)	5 (10%)	4 (13%)	0.9
**Selected Dietary Recommendations: Increase fruits and vegetables**	32 (76%)	39 (80%)	24 (75%)	0.9
**Selected Dietary Recommendations: Doesn't matter, eat what you want**	9 (21%)	3 (6.1%)	11 (34%)	**0.005**
**Selected Dietary Recommendations: Minimize/avoid processed meat**	20 (48%)	31 (63%)	13 (41%)	0.11
**Selected Dietary Recommendations: Minimize/avoid red meat**	16 (38%)	24 (49%)	8 (25%)	0.10
**Selected Dietary Recommendations: Increase whole grains**	21 (50%)	26 (53%)	11 (34%)	0.2
**Selected Dietary Recommendations: Increase beans and other legumes**	14 (33%)	25 (51%)	11 (34%)	0.2
**Selected Dietary Recommendations: Minimize/avoid sugary beverages**	20 (48%)	31 (63%)	15 (47%)	0.2
**Selected Dietary Recommendations: Minimize/avoid processed food**	25 (60%)	32 (65%)	13 (41%)	0.083
**Selected Dietary Recommendations: Eat high calorie foods or whatever you like to avoid losing weight**	17 (40%)	13 (27%)	7 (22%)	0.2
**Selected Dietary Recommendations: Eat more animal protein**	5 (12%)	3 (6.1%)	1 (3.1%)	0.4

Characteristic	Predominant Dietary Pattern Omnivore N = 42[1]	Pescatarian / Vegetarian / Vegan / Mediterranean N = 49[1]	Western N = 32[1]	p-value[2]
**Selected Dietary Recommendations: Eat more plant protein**	11 (26%)	9 (18%)	2 (6.3%)	0.085
**Selected Dietary Recommendations: Eat low carbohydrate foods**	9 (21%)	9 (18%)	1 (3.1%)	**0.049**
**Selected Dietary Recommendations: Eat more dairy**	4 (9.5%)	4 (8.2%)	2 (6.3%)	>0.9
**Selected Dietary Recommendations: Consider intermittent fasting**	2 (4.8%)	3 (6.1%)	1 (3.1%)	>0.9
**Selected Dietary Recommendations: Eat foods to reduce weight to an ideal body weight**	6 (14%)	5 (10%)	1 (3.1%)	0.3
**Selected Dietary Recommendations: Eat more protein of any type (animal or plant)**	12 (29%)	21 (43%)	11 (34%)	0.4
**Selected Dietary Recommendations: Minimize dairy intake**	3 (7.1%)	4 (8.2%)	0 (0%)	0.3
**Selected Dietary Recommendations: Other (please specify):**	2 (4.8%)	3 (6.1%)	2 (6.3%)	>0.9
**It is the role of an oncologist/hematologist to discuss nutrition -**				**0.024**
a. Strongly agree	10 (24%)	18 (38%)	5 (16%)	
b. Agree	20 (48%)	25 (53%)	17 (53%)	
c. Neither agree nor disagree	11	2 (4.3%)	8	

Characteristic	Predominant Dietary Pattern<br>Omnivore<br>N = 42[1]	 Pescatarian / Vegetarian / Vegan / Mediterranean<br>N = 49[1]	 Western<br>N = 32[1]	p-value[2]
	(26%)		(25%)	
d. Disagree	1 (2.4%)	2 (4.3%)	2 (6.3%)	
Unknown	0	2	0	
**Selected Dietary Patterns for Cancer Prevention/Treatment/Survivorship: Plant-based diets (predominantly unprocessed plant-based foods; may or may not include small amounts of animal products)**	19 (45%)	22 (45%)	17 (53%)	0.7
**Selected Dietary Patterns for Cancer Prevention/Treatment/Survivorship: There is not enough evidence to recommend any dietary pattern**	14 (33%)	17 (35%)	12 (38%)	>0.9
**Selected Dietary Patterns for Cancer Prevention/Treatment/Survivorship: Mediterranean**	21 (50%)	28 (57%)	19 (59%)	0.7
**Selected Dietary Patterns for Cancer Prevention/Treatment/Survivorship: Low carbohydrate diets, including ketogenic and Paleo**	3 (7.1%)	5 (10%)	3 (9.4%)	>0.9
**Selected Dietary Patterns for Cancer Prevention/Treatment/Survivorship: Pescatarian**	6 (14%)	4 (8.2%)	2 (6.3%)	0.5
**Selected Dietary Patterns for Cancer Prevention/Treatment/Survivorship: Other (please specify):**	3 (7.1%)	2 (4.1%)	0 (0%)	0.4
**Selected Training on Nutrition and Cancer: No, I have not received specific training,**	13	11 (22%)	15	0.069

Characteristic	Predominant Dietary Pattern<br>Omnivore<br>N = 42[1]	 Pescatarian / Vegetarian / Vegan / Mediterranean<br>N = 49[1]	 Western<br>N = 32[1]	p-value[2]
**nor have I spent time reading about this topic**	(31%)		(47%)	
**Selected Training on Nutrition and Cancer: No, I taught myself through reading books or journal articles**	21 (50%)	27 (55%)	14 (44%)	0.6
**Selected Training on Nutrition and Cancer: Yes, I received formal training during medical school**	4 (9.5%)	2 (4.1%)	2 (6.3%)	0.5
**Selected Training on Nutrition and Cancer: Yes, I received formal training during residency**	3 (7.1%)	2 (4.1%)	2 (6.3%)	0.8
**Selected Training on Nutrition and Cancer: Yes, I received formal training during oncology training**	6 (14%)	2 (4.1%)	0 (0%)	**0.036**
**Selected Training on Nutrition and Cancer: Yes, I have taken an accredited course or attended a conference on nutrition and health (If yes, please specify course/conference)**	0 (0%)	6 (12%)	0 (0%)	**0.010**
**How important or unimportant are dietary choices for patients with cancer in the following situations? - Risk Reduction/Pre-diagnosis**				**0.024**
Not sure/Neutral	2 (4.8%)	2 (4.3%)	3 (9.4%)	
Somewhat important	11 (26%)	7 (15%)	15 (47%)	
Somewhat unimportant	1	1 (2.1%)	2	

Characteristic	Omnivore N = 42[1]	Pescatarian / Vegetarian / Vegan / Mediterranean N = 49[1]	Western N = 32[1]	p-value[2]
	**Predominant Dietary Pattern**			
	(2.4%)		(6.3%)	
Very important	27 (64%)	36 (77%)	12 (38%)	
Very unimportant	1 (2.4%)	1 (2.1%)	0 (0%)	
Unknown	0	2	0	
**How important or unimportant are dietary choices for patients with cancer in the following situations? - Risk Reduction/Pre-diagnosis (Very Important vs Other Choice)**	27 (64%)	36 (77%)	12 (38%)	**0.002**
Unknown	0	2	0	
**How important or unimportant are dietary choices for patients with cancer in the following situations? - During curative treatment**				**0.013**
Not sure/Neutral	5 (12%)	6 (13%)	6 (19%)	
Somewhat important	15 (36%)	6 (13%)	12 (38%)	
Somewhat unimportant	2 (4.8%)	2 (4.3%)	4 (13%)	
Very important	17 (40%)	32 (68%)	10 (31%)	
Very unimportant	3 (7.1%)	1 (2.1%)	0 (0%)	

Characteristic	Predominant Dietary Pattern<br>Omnivore<br>N = 42[1]	 Pescatarian / Vegetarian / Vegan / Mediterranean<br>N = 49[1]	 Western<br>N = 32[1]	p-value[2]
Unknown	0	2	0	
**How important or unimportant are dietary choices for patients with cancer in the following situations? - During curative treatment (Very Important vs Other Choice)**	17 (40%)	32 (68%)	10 (31%)	**0.002**
Unknown	0	2	0	
**How important or unimportant are dietary choices for patients with cancer in the following situations? - Post treatment survivorship/relapse prevention**				0.12
Not sure/Neutral	2 (4.8%)	2 (4.3%)	2 (6.3%)	
Somewhat important	12 (29%)	10 (21%)	17 (53%)	
Somewhat unimportant	1 (2.4%)	2 (4.3%)	1 (3.1%)	
Very important	25 (60%)	32 (68%)	12 (38%)	
Very unimportant	2 (4.8%)	1 (2.1%)	0 (0%)	
Unknown	0	2	0	
**How important or unimportant are dietary choices for patients with cancer in the following situations? - Post treatment survivorship/relapse prevention (Very**	25 (60%)	32 (68%)	12 (38%)	**0.024**

Characteristic	Predominant Dietary Pattern<br>Omnivore<br>N = 42[1]	 Pescatarian / Vegetarian / Vegan / Mediterranean<br>N = 49[1]	 Western<br>N = 32[1]	p-value[2]
**Important vs Other Choice)**				
Unknown	0	2	0	
**How important or unimportant are dietary choices for patients with cancer in the following situations? - During palliative treatment**				**0.013**
Not sure/Neutral	7 (17%)	5 (11%)	4 (13%)	
Somewhat important	4 (9.5%)	12 (26%)	10 (31%)	
Somewhat unimportant	8 (19%)	3 (6.4%)	7 (22%)	
Very important	19 (45%)	25 (53%)	6 (19%)	
Very unimportant	4 (9.5%)	2 (4.3%)	5 (16%)	
Unknown	0	2	0	
**How important or unimportant are dietary choices for patients with cancer in the following situations? - During palliative treatment (Very Important vs Other Choice)**	19 (45%)	25 (53%)	6 (19%)	**0.008**
Unknown	0	2	0	
**Based on available evidence at this time do you believe there is enough information to provide nutrition**				0.4

Characteristic	Predominant Dietary Pattern<br>Omnivore<br>N = 42[1]	 Pescatarian / Vegetarian / Vegan / Mediterranean<br>N = 49[1]	 Western<br>N = 32[1]	p-value[2]
**recommendations to your patients for the following: - Reduce comorbidities (such as obesity, diabetes, cardiovascular disease) to improve survival.**				
No	1 (2.4%)	5 (11%)	3 (9.4%)	
Unsure	2 (4.8%)	5 (11%)	3 (9.4%)	
Yes	39 (93%)	37 (79%)	26 (81%)	
Unknown	0	2	0	
**Based on available evidence at this time do you believe there is enough information to provide nutrition recommendations to your patients for the following: - Improve quality of life**				0.3
No	3 (7.1%)	5 (11%)	7 (22%)	
Unsure	8 (19%)	5 (11%)	6 (19%)	
Yes	31 (74%)	37 (79%)	19 (59%)	
Unknown	0	2	0	
**Based on available evidence at this time do you believe there is enough information to provide nutrition recommendations to your patients for the following: - Improve cancer treatment**				0.7

Characteristic	Omnivore N = 42[1]	Pescatarian / Vegetarian / Vegan / Mediterranean N = 49[1]	Western N = 32[1]	p-value[2]
	Predominant Dietary Pattern			
**response**				
No	18 (43%)	20 (43%)	16 (50%)	
Unsure	8 (19%)	8 (17%)	8 (25%)	
Yes	16 (38%)	19 (40%)	8 (25%)	
Unknown	0	2	0	
**Based on available evidence at this time do you believe there is enough information to provide nutrition recommendations to your patients for the following: - Improve cancer-specific survival endpoints such as progression-free survival and overall survival**				0.5
No	18 (43%)	21 (45%)	16 (50%)	
Unsure	9 (21%)	10 (21%)	10 (31%)	
Yes	15 (36%)	16 (34%)	6 (19%)	
Unknown	0	2	0	

[1] n (%)

[2] Fisher's Exact Test for Count Data with simulated p-value (based on 2000 replicates); Pearson's Chi-squared test
